# The relation between speaking-style categorization and speech recognition in adult cochlear implant users

**DOI:** 10.1121/10.0017439

**Published:** 2023-03

**Authors:** Terrin N. Tamati, Esther Janse, Deniz Başkent

**Affiliations:** 1 Department of Otolaryngology–Head and Neck Surgery, Vanderbilt University Medical Center, Nashville, Tennessee 37232, USA; 2 Centre for Language Studies, Radboud University Nijmegen, Nijmegen, The Netherlands; 3 Department of Otorhinolaryngology/Head and Neck Surgery, University Medical Center Groningen, University of Groningen, The Netherlands

## Abstract

The current study examined the relation between speaking-style categorization and speech recognition in postlingually deafened adult cochlear implant users and normal-hearing listeners tested under 4- and 8-channel acoustic noise-vocoder cochlear implant simulations. Across all listeners, better speaking-style categorization of careful read and casual conversation speech was associated with more accurate recognition of speech across those same two speaking styles. Findings suggest that some cochlear implant users and normal-hearing listeners under cochlear implant simulation may benefit from stronger encoding of indexical information in speech, enabling both better categorization and recognition of speech produced in different speaking styles.

## 1. Introduction

Talkers may enhance the clarity of their speech through hyperarticulating or exaggerating sound segments and syllables, increasing loudness, or slowing their speaking rate (e.g., Hazan et al., 2018; Krause and Braida, 2002, 2004). In contrast, conversational speech is characterized by hypoarticulated speech in which entire sound segments or syllables may be reduced, resulting in, for example, a smaller vowel space as well as a faster speaking rate (e.g., Ernestus and Warner, 2011). Clear speech modifications typically result in an intelligibility benefit relative to conversational speech, particularly if reduced words are presented in isolation (e.g., Janse et al., 2007; Ranbom and Connine, 2007); in challenging conditions, such as in noise or babble (e.g., Helfer, 1997, 1998; Schum, 1996); or with a hearing impairment (Janse and Ernestus, 2011). For many adults with cochlear implants (CIs), conversational speech may be particularly challenging, leading to a benefit for clear speech even in quiet conditions (Iverson and Bradlow, 2002; Liu et al., 2004). However, CI users’ ability to recognize clear or conversational speech may depend on the extent to which they are able to perceive and make use of detailed speaking-style cues. The current study examined the relation between the recognition of speech produced in different speaking styles and speaking-style categorization across CI users and normal-hearing (NH) listeners under CI simulation.

Implant transmitted speech signals are heavily reduced in acoustic-phonetic details compared to normal acoustic hearing, due to the limitations in information transmission of electric stimulation of the auditory nerve [for a review, see Başkent et al. (2016b)]. Although CI users and NH listeners under CI simulation broadly benefit from clear speech, the recognition of speech across speaking styles has been shown to vary across individual CI and NH listeners (Liu et al., 2004; Rodman et al., 2020). Results from these studies have shown that the recognition of clear and conversational speech depends on baseline speech intelligibility in CI users and spectral resolution—determined by the number of spectral channels implemented in the simulations—in NH listeners. Thus, the ability to recognize clear and conversational speech may vary across individuals, determined at least in part by auditory spectral resolution.

The ability to adapt to and recognize speaking style may be related to individual CI users’ access to and ability to make use of detailed speaking-style cues. Previous studies have demonstrated an association between better discrimination of talker voice and accent details and more accurate word-in-sentence recognition in both NH listeners (Tamati et al., 2013) and CI users (Tamati et al., 2021), suggesting that some CI and NH listeners may benefit from better encoding of fine acoustic-phonetic details (Tamati et al., 2013). Individual differences in the perception of talker voice and accent variability have been observed in CI users (Cleary and Pisoni 2002; Hay-McCutcheon et al., 2018; Tamati et al., 2021), which may be related to both auditory sensitivity and cognitive-linguistic ability (Li et al., 2022). For speaking style, our previous findings also demonstrated individual variability in speaking-style categorization among NH listeners with unprocessed and 12- and 4-channel noise-vocoded speech (Tamati et al., 2019). Similarly, some CI users may also benefit from stronger encoding of pronunciation details to categorize speaking styles and facilitate speech recognition across speaking styles, potentially due to both individual differences in access to detailed speaking-style cues and individual differences in adaptation or compensation mechanisms required to make use of this information. However, the relation between word-in-sentence recognition across speaking styles and (across talker) speaking-style categorization has not yet been established.

The current study investigated the relation between speaking-style categorization and speech recognition across adult CI users and NH listeners under CI simulation, using a speaking-style categorization task and a word-in-sentence recognition task. Both tasks involved the use of two speaking styles, specifically produced in a read list (“careful read”) and speech produced in the context of a conversation (“casual conversation”). These speaking styles were selected since they contain features consistent with clear and conversational speech, respectively (Tamati et al., 2019). NH listeners completed the tasks either under conditions of low (4-channel; CI-4) or high spectral resolution (8-channel; CI-8) acoustic noise-vocoder simulations of CI hearing [based on the wide range of speech recognition performance roughly matching the range of CI performance, e.g., Friesen et al. (2001) and Gaudrain and Başkent (2018)]. Given previous findings demonstrating a relation between the perception of indexical variability and word recognition in CI users and NH listeners (e.g., Tamati et al., 2013; Tamati et al., 2021), we expected that speaking-style categorization and word-in-sentence recognition would be associated across all listeners. This finding would suggest that both bottom-up spectral resolution and top-down factors, potentially related to adaptation or compensation mechanisms, contribute to overall performance on the two speech perception tasks and to the relation between performance on the tasks. Alternatively, performance on the tasks may not be associated across all listeners if spectral resolution is the only factor contributing to performance on both speech perception tasks; in that case, then we would expect NH participants within each group to show similar performance levels (on both tasks). Another alternative is that the relation between performance on the two tasks may differ across listener groups. If performance on one of the tasks (for example, word-in-sentence recognition) relies more heavily on cognitive mechanisms for one listener group, because the use of cognitive mechanisms is contingent upon either spectral resolution (NH 4- vs 8-channel) or hearing status (CI vs NH listeners), then the relation between the two tasks would differ across listener groups. Therefore, the current study examined whether performance on the word-in-sentence recognition task and speaking-style categorization task is related across CI and NH listeners under CI simulation and explored whether the strength of the relation differs by listener group.

## 2. Methods

### 2.1 Listeners

CI users included ten native Dutch CI users [age 38–75 years; mean (*M*) = 68, standard deviation (*SD*) = 11.3; 3 female], with more than 2.5 years of CI use (2.5–13 years). CI users used their everyday CI map set to a comfortable volume to ensure optimal audibility. Additional participant demographics are provided in Table 1.

NH listeners included 20 young, native, NH Dutch speakers (age 20–29 years; *M* = 20.6; *SD* = 1.5; 15 female), with hearing thresholds ≤ 25 dB hearing level (HL) from 250 to 8000 Hz. NH participants were randomly divided into vocoder simulated higher- (CI-8) or lower-spectral resolution (CI-4) groups.

### 2.2 Materials

Materials consisted of 144 unique sentence-length utterances from six talkers (3 female/3 male; all native speakers of Dutch, 20–66 years old) from the Instituut voor Fonetische Wetenschappen Amsterdam (IFA) corpus (Van Son et al., 2001). The same set of utterances was used in both the word-in-sentence recognition and speaking-style categorization tasks. Seventy-two sentence-length utterances were from a read list (careful read), 12 × 6 talkers, and 72 sentence-length utterances were produced in the context of an informal conversation with an interviewer (casual conversation), 12 × 6 talkers. Therefore, in total, each talker produced 24 utterances (12 careful read and 12 casual conversation). As described in Tamati et al. (2019), utterances were selected to minimize differences in semantic content, number of words, and pauses across speaking styles. For a detailed description of the acoustic properties of a larger set of materials from which the stimuli were selected, see Tamati et al. (2019). As described in Tamati et al. (2019), the careful read speech of the larger corpus demonstrated properties consistent with a clear speaking style, including slightly slower speaking rate (although varying across talkers), a higher average *F*0 and *F*0 range, and more fully realized sound segments (e.g., more frequent word-final [t]-realization, schwa realization in unstressed syllables, word-final [n]-realization, and postvocalic-[r] realization). In contrast, the casual conversation speech displayed more characteristics of conversational speech, including a slightly faster speaking rate, a lower average *F*0 and *F*0 range, and more frequent reduction/deletion of sound segments. Overall, the two speaking styles in the current study are consistent with descriptions among scripted and variations of nonscripted speech in Dutch (Ernestus et al., 2015).

The CI-simulation conditions were made by a noise-band vocoder using matlab code maintained by the dB SPL at the University Medical Center Groningen (Gaudrain, 2016). The original stimuli (for envelopes) and a white noise (for carrier) were filtered into eight (CI-8) or four (CI-4) frequency bands between 150 and 7000 Hz, using 12th-order, zero-phase Butterworth filters, corresponding to even cochlear spacing using Greenwood’s frequency-to-place mapping function (Greenwood, 1990). From each frequency band of speech signal, the temporal envelope was extracted by half-wave rectification and low-pass filtering with a cutoff frequency of 300 Hz, using a zero-phase 4th-order Butterworth filter. The stimuli were constructed by modulating the noise carrier bands in each channel with the corresponding extracted envelope from speech band. The envelope modulated noise bands from all vocoder channels were added together to produce the final stimuli.

### 2.3 Procedure

Participants completed the word-in-sentence recognition task followed by the speaking-style categorization task. Before completing these tasks, all participants received examples of careful read and casual conversation speech from written instructions as well as live voice and pre-recorded examples to reinforce the written instructions. NH listeners also listened to a vocoded version of the North Wind and the Sun passage in Dutch (“De noordenwind en de zon”) at the beginning of the study to familiarize them with the degraded sound quality of noise-vocoded speech. Stimuli were presented at 65 dB SPL, via a loudspeaker placed approximately 1 m from the participant at 0° azimuth.

#### 2.3.1 Word-in-sentence recognition task

Participants were presented with a single stimulus and repeated the words that they heard, as quickly as possible. Partial answers and guessing were encouraged. All 144 stimuli (72 careful read and 72 casual conversation) were randomly presented within the same block. Stimulus items were not repeated within the word-in-sentence recognition task but were later presented again in the speaking-style categorization task. The task was self-paced, and participants could move to the next trial when ready. Responses were recorded and scored offline by a native Dutch speaker for words correctly identified. Exact word order was not required, but plural or possessive morphological markers were required to match the word. The percentage of the total number of words correctly recognized, including all content and function words, was calculated.

#### 2.3.2 Speaking-style categorization task

Participants were presented with a single stimulus and indicated whether it was produced in a formal (or clear) manner or an informal (or conversational) manner, placing special focus on *how* the utterance was said and not on *what* was said. Participants were encouraged to respond as quickly as possible, but they could take as much time as they wanted before moving on to the next trial. The same 144 stimulus items as in the word-in-sentence recognition task were randomly presented in the speaking-style categorization task, without repeat. To account for potential listener bias in responses, *d*′ (*d*-prime) scores, which incorporate both the identification rate (hits) and the false alarm rates, were calculated (Macmillan and Creelman, 2005).

## 3. Results

**Fig. 1.**
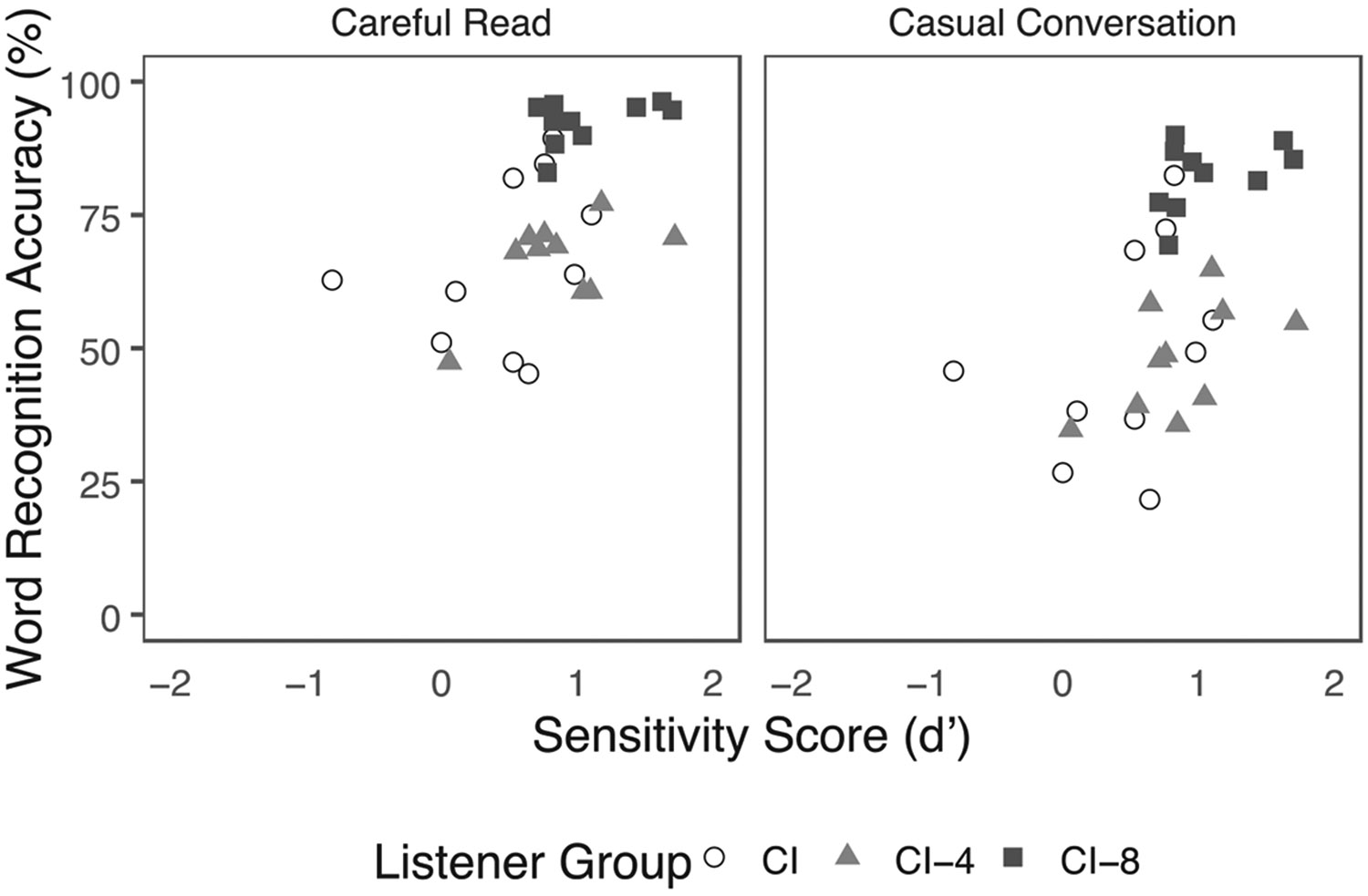
Scatterplots demonstrating sensitivity scores (*d*′) in the speaking-style categorization task (*x* axis) and mean percent word-in-sentence recognition accuracy [careful read (left); casual conversation (right)] (*y* axis), for CI users (open circles), CI-4 (filled light gray triangles), and CI-8 (filled dark gray squares).

A multivariate linear regression analysis was carried out with word-in-sentence recognition accuracy for careful read and casual conversation sentences—in rational arcsine units (RAUs; Studebaker, 1985)—as the dependent variables and categorization performance (*d*′ scores) as the predictor variable. This analysis was chosen to simultaneously assess the relation between categorization performance and the recognition of careful read and casual conversation speech and to evaluate whether the relation between categorization and recognition exists for both speaking styles (Fig. 1). Overall, better categorization performance (*d*′ scores) across individuals in all three listening groups was significantly associated with more accurate recognition of both careful read [*R*^2^ = 0.26, *F*(1,28) = 9.74, *p* = 0.004] and casual conversation sentences [*R*^2^ = 0.22, *F*(1,28) = 7.71, *p* < 0.010]. For each 1-unit increase in *d*′ score, listeners obtain an 18.3 and 19.2 increase in accuracy for careful read and casual conversation sentences (in RAU), respectively.

To further explore whether the relation between word-in-sentence recognition accuracy and categorization performance (*d*′ scores) varied by listener group, an analysis of covariance (ANCOVA) was conducted on overall recognition accuracy (both careful read and casual conversation sentences) as the dependent variable with categorization performance (*d*′ scores), listener group (CI, CI-4, CI-8), and the interaction between categorization performance and listener group as the predictor variables. For this analysis, overall recognition accuracy was used since categorization performance appeared to relate to recognition accuracy for both careful read and casual conversation sentences. Categorization task performance (*d*′ scores) had a significant effect on overall word-in-sentence recognition accuracy [*F*(1,24) = 19.88, *p* < 0.001]. After accounting for categorization performance, listener group also had a significant effect on overall word-in-sentence recognition accuracy [*F*(2,24) = 12.84, *p* < 0.001]. However, the interaction between categorization task performance and listener group was not significant, suggesting that the relation between word-in-sentence recognition accuracy and categorization performance (*d*′ scores) did not vary by listener group.

## 4. Discussion

The current study investigated the relation between the categorization and recognition of speech produced in two speaking styles across CI users and NH listeners under 4- and 8-channel acoustic noise-vocoder cochlear implant simulations. Consistent with our main hypothesis that speaking-style categorization would be related to word-in-sentence recognition accuracy across all listeners, we observed that individual listeners who showed better categorization of speaking styles also showed more accurate word-in-sentence recognition for both careful and casual speech. This finding is consistent with previous studies that have observed an association between word-in-sentence recognition accuracy (across a range of adverse conditions), on the one hand, and the perception of indexical variability, on the other hand, including regional accent categorization in NH listeners (Tamati et al., 2013), foreign-accent discrimination in pre-lingually deafened and implanted children and adults with CIs (Tamati et al., 2021), and talker discrimination in pre-lingually deafened children with CIs (Cleary and Pisoni, 2002). The findings from the current study extend these results by demonstrating this relation for the perception of speaking style and across listener groups, including adult CI users and NH listeners under CI simulation. Taken together with previous findings, these results suggest that the processing of linguistic and indexical properties of speech appears to be closely coupled across the lifespan in children and adults with and without hearing impairment.

We further examined this relation by listener group (CI users and NH listeners under CI simulation) to assess the factors underlying the relation between speech recognition and speaking-style categorization. Overall, word-in-sentence recognition was less accurate for the vocoder simulated low-spectral resolution group (CI-4) than the vocoder simulated high-spectral resolution group (CI-8), and (although not directly assessed here) *d*′ scores were better for the CI-8 listener group than the CI-4 group, as displayed in Fig. 1. These findings suggest that spectral resolution determines, at least in part, both speech recognition across speaking styles and speaking-style categorization.

As a group, CI users performed more similarly to the CI-4 listener group on both tasks, but individual CI users were spread across the range of scores of both CI-4 and CI-8 listeners. Although it cannot be determined in the current study, this group of CI users may have varied in their basic auditory sensitivity (e.g., spectral and temporal resolution), contributing to variability in performance on the two tasks. CI-8 listeners and some individual CI users (who fall within the performance range of the CI-8 listeners) may have better access to reliable speaking-style cues, compared to CI-4 listeners, conveyed by spectral properties of speech sounds. CI users, and NH listeners under CI simulation, may also be able to rely to some extent upon gross temporal cues, such as speaking rate differences (Tamati et al., 2019). Additionally, differences in lexical content and/or the presence of pauses, disfluencies, speech errors, or informal words could potentially be used as speaking-style cues (e.g., Bradlow and Bent, 2003; Schuppler et al., 2011). However, lexical content was roughly matched across speaking styles, and the other potential cues occurred infrequently (Tamati et al., 2019). Overall, the results are consistent with previous studies using acoustic vocoder simulations of CI hearing demonstrating reduced speech recognition with decreasing spectral resolution (i.e., with decreasing number of spectral channels) (e.g., Fu et al., 1998; Friesen et al., 2001) and support prior studies demonstrating that intelligibility differences between clear and conversational speech (here, careful read and casual conversation) emerge in degraded conditions, such as in background noise or babble or with hearing impairment (e.g., Payton et al., 1994; Janse and Ernestus, 2011).

Although there is clearly an overall effect of spectral resolution, individual differences on both tasks emerged within those groups despite experiencing similar levels of signal degradation (via specific CI simulations where all parameters are well controlled). Additionally, the relation between word-in-sentence recognition and speaking-style categorization appears to be similar for the two NH listener groups, regardless of overall performance levels induced by manipulations of signal degradation (8- or 4-channel CI simulation), and for CI users. These findings suggest that listeners may vary in the cognitive compensation strategies that they use—regardless of overall signal degradation or hearing status—to both categorize speaking style and recognize spectrotemporally degraded speech. Previous research suggests that some individual CI users may be able to more actively use cognitive resources to enhance the processing of degraded speech (e.g., Başkent et al., 2016a; Heydebrand et al., 2007). Similarly, individual CI users who have relatively greater access to detailed acoustic-phonetic information and/or more effective use of cognitive mechanisms may benefit from stronger encoding of fine acoustic-phonetic details in degraded speech to discriminate different sources of variability and understand real-life challenging speech.

We have noted some points of our study that can be improved in future research. First, we would have ideally liked to have had a larger number of participants within each group; in the current study, sample sizes of each group were relatively small with only ten participants. Second, although our study was not aimed at directly comparing group performance levels, the CI users and NH listeners differed in age and likely other demographic, auditory, and cognitive factors that may contribute to performance and possibly in different ways for each task (e.g., Bhargava et al., 2016). Additionally, CI users show a large age range and diverse language background and experiences, related to age at implantation, duration of deafness prior to implantation, duration of CI use, and use of hearing aids, among other factors that influence speech recognition outcomes (e.g., Blamey et al. 2013; Heydebrand et al., 2007). These group and individual factors could be better controlled or directly investigated in future studies. Third, NH performance with unprocessed speech could be assessed. This group was not included in the current study since the recognition of the careful read and casual conversation speech is near or at ceiling for NH listeners (Tamati et al., 2019). However, speaking-style categorization is more challenging but still above chance for NH listeners with unprocessed speech (Tamati et al., 2019). Obtaining sensitivity scores for unprocessed speech would facilitate the broader interpretation of the factors involved in speaking-style categorization across listener populations, including stimulus-related factors. Future research, possibly on larger CI user populations, multi-center studies, and/or longitudinal studies, is needed to better understand the individual factors that impact the perception of real-life speech variability in adult CI users.

## 5. Conclusion

The current study investigated individual differences in the perception of speaking styles in CI users and NH listeners in vocoder simulated high- and low-spectral resolution groups (8- and 4-channel acoustic noise-vocoder simulations, respectively). The results of this study demonstrated that individual CI and NH listeners who showed better speaking-style categorization, specifically between careful read and casual conversation speech, also showed more accurate overall word-in-sentence recognition across the same two speaking styles. These findings suggest that some CI users and NH listeners under CI simulation may benefit from stronger encoding of detailed acoustic-phonetic information to categorize speaking styles and recognize speech. Moreover, these listeners may have a greater ability to take advantage of speaking-style cues to facilitate speech recognition, if signal quality affords access to these cues or if cognitive compensation mechanisms can be effectively used, potentially providing them with an advantage in real-life conditions.

## Figures and Tables

**Table 1.** Demographic information for CI users.

Participant	Age	Gender^a^	Etiology	Age at onset of hearing loss (years)	Duration of CI use (years)	Device	Implant side	Better ear PTA^b^ (dB HL)	Hearing aid
CI1	67	M	Genetic-progressive	13	3	AB HiRes 90K Helix (Harmony)	Left	95	No
CI2	75	M	Traumatic head injury	68	8	Cochlear CI24R CS (Freedom)	Right	120	No
CI3	78	F	Unknown	0	10	Cochlear CI24R CS (Freedom)	Right	120	No
CI4	68	M	Autoimmune	29	10	Cochlear CI24R CS (Freedom)	Left	120	No
CI5	75	M	Genetic-progressive	50	9	AB HiRes 90K Helix (Harmony)	Bilateral	120	NA^c^
CI6	68	M	Viral-sudden	61	6	Cochlear CI24R CS (Freedom)	Right	120	No
CI7	66	F	Unknown-progressive	34	2.5	AB HiRes 90K Helix (Harmony)	Right	96.25	No
CI8	38	M	Meningitis	1	13	Cochlear CI24R CS (Freedom)	Right	120	Yes
CI9	70	M	Unknown	55	3	AB HiRes 90K Helix (Harmony)	Right	68.75	Yes
CI10	60	F	Genetic-progressive	17	13	Cochlear CI24R CS (Freedom)	Right	113.75	Yes

a Gender as reported by the participant.

b Unaided pure tone average (PTA) across 0.5, 1, 2, and 4 kHz.

c Not applicable (NA).
